# Influence of Nanoparticle Pretreatment on the Thermal, Rheological and Mechanical Properties of PLA-PBSA Nanocomposites Incorporating Cellulose Nanocrystals or Montmorillonite

**DOI:** 10.3390/nano9010029

**Published:** 2018-12-26

**Authors:** Wissam Abdallah, Amin Mirzadeh, Victor Tan, Musa R. Kamal

**Affiliations:** Department of Chemical Engineering, McGill University, Montreal, QC H3A 0C5, Canada;; wabdallah@outlook.com (W.A.); amin.mirzadeh@novachem.com (A.M.); victor.tan@mcgill.ca (V.T.)

**Keywords:** spray freeze-drying, poly(lactic acid), poly[(butylene succinate)-*co*-adipate], cellulose nanocrystals, montmorillonite, nanocomposite

## Abstract

Nanoparticles based on cellulose nanocrystals (CNC) and montmorillonite clay (MMT) were prepared using spray freeze-drying. The nanoparticles were then used as reinforcement to prepare nanocomposites with poly(lactic acid) (PLA) as the polymer matrix. The effect of spray freeze-dried CNC (SFD-CNC) and spray freeze-dried MMT (SFD-MMT) on the rheological and mechanical properties of PLA and its blends with poly[(butylene succinate)-*co*-adipate)] (PBSA) were investigated. An epoxy chain extender was used during preparation of the blends and nanocomposites to enhance the mechanical properties of the products. Different methods such as scanning electron microscopy, X-ray diffraction and adsorption/desorption analyses were used to characterize the prepared nanoparticles and their localization in the blends. Dynamic oscillatory shear behavior, elongational viscosity and mechanical characteristics of the nanocomposites of PLA and the blends were evaluated. The results obtained for nanocomposites filled with unmodified SFD-MMT were compared with those obtained when the filler was a commercial organically modified montmorillonite nanoclay (methyl-tallow-bis(2-hydroxyeethyl) quaternary ammonium chloride) (C30B), which was not spray freeze-dried.

## 1. Introduction

Biosource, biodegradable and ecofriendly thermoplastic polymers are of interest in the fabrication of sustainable nanocomposites [1]. Poly(lactic acid) (PLA) is an environmentally benign biopolymer derived from renewable resources (i.e. wheat, corn, rice). It is biodegradable, recyclable, compostable and biocompatible [2]. Thus, it can be used in various biomedical, pharmaceutical and agricultural applications [3,4]. PLA hydrolyzes to its constituent hydroxyl acid when implanted in living organisms and its degradation products are nontoxic [5]. Moreover, the energy consumption cost to produce PLA is 25–55% of the corresponding cost for petroleum-based polymers [3]. It can be processed by injection molding, film extrusion, blow molding, thermoforming, fiber spinning and film forming by virtue of its good thermal processability compared to other biopolymers [3].

In spite of all its attractive features, PLA has some drawbacks, which significantly limit its expansion into some commercial application areas. PLA has low glass transition temperature (Tg), and it exhibits poor toughness with less than 10% elongation at break, poor thermal stability, low-heat distortion temperature and weak barrier properties [4,6]. Thus, it cannot be used in applications that require plastic deformation at higher stress levels (e.g., screws and fracture repair plates). Moreover, PLA degrades slowly via the hydrolysis of backbone ester groups, which limits its usage in some biomedical applications. In some important polymer processing operations such as fiber spinning, blow molding, and film production, the utilization of PLA is limited because of its low melt strength [3].

It has been reported that some of the above shortcomings, particularly the low melt strength of PLA and its brittleness in the solid state, may be mitigated by using blends based on PLA with poly[(butylene succinate)-*co*-adipate)] (PBSA) as well as their nanocomposites with nanoclay [6]. This approach takes advantage of the low glass transition temperature and high ductility and melt strength of rubbery PBSA. Significant strain hardening behavior of the melt was observed for blends containing 50 wt. % or higher PBSA content. However, the effect of clay on the elongational viscosity, the melt strength and mechanical properties of the blends and PLA/PBSA/clay ternary nanocomposites were rather small. Ojijo et al. [7] showed the significance of the effect of clay content and localization on the properties of PLA/PBSA (70/30) composites containing organoclay (0 to 9 wt. %) prepared via melt compounding in a batch mixer. Clay had slightly better dispersion in PBSA than in PLA, and there was a tendency of the silicate layers to delaminate in PBSA at low clay content. Composite with 2 wt. % clay content showed slight improvement in elongation at break and yielded the optimum properties overall. Gui and coworkers [8] studied the effect of the blend ratios on the morphology and melt rheology of PLA/PBSA blends. Interfacial tensions were calculated by fitting the complex moduli using the Palierne model and weighted relaxation spectra evaluated from linear viscoelastic data. The increase in the storage modulus, in the low frequency region was more distinct in PLA/PBSA blends than in their pure components, whereas at PBSA content higher than 20%, a second plateau was observed. Weighted relaxation spectra showed that there was a longer relaxation time for the blend system.

Cellulose nanocrystals (CNC), individual cellulose crystallites that form the building blocks for cellulose structures in plants, have attracted significant interest recently as a green nano-reinforcing agent in polymer-based composites [9]. Cellulose nanocrystals can be conveniently extracted from the bulk amorphous cellulosic phase by mechanical or chemical treatment [10]. On the other hand, layered montmorillonite silicates, the most common mineral of the smectite group, is used as reinforcement for polymer nanocomposites by virtue of its high aspect ratio, morphology, natural abundance, ecological nature, and low cost. It is a naturally occurring 2:1 phyllosilicate with a high surface area of approximately 750 m^2^/g, and its crystal lattice is composed of two silica tetrahedral sheets (SiO_4_) and an octahedral alumina sheet (AlO_4_(OH)_4_) between the tetrahedrons [11]. To facilitate the intercalation of MMT clay with polymers in nanocomposites, the wetting properties of polymer-MMT interface need to be enhanced by modifying the interfacial surface energy with the polymer and enlarging the MMT basal spacing [12]. This can be achieved by ion exchange of the hydrated ions present in MMT with cationic surfactants, which in turn renders the hydrophilic nature of MMT silicates organophilic. 

The incorporation of biosource, biocompatible, and biodegradable cellulosic nanoparticles into biodegradable blends could present advantages for nanocomposites that exhibit biodegradability and sustainability, in addition to potential enhancement of processing and mechanical properties. Usually, due to the hydrophilic nature of CNC, nanocomposites are prepared using water suspensions of CNC [13,14,15]. However, melt processing of polymer-based nanocomposites is more desirable, due to environmental and energy consumption considerations and lower processing equipment costs. Melt processing of the nanocomposites is utilized in the present work.

The dispersibility of dried CNC powder in water is a critical requirement for producing colloidal suspensions. This can be achieved by ion exchange (from acidic form to neutral sodium-form), the use of additives [16] and large energy input (e.g. shear mixing, sonication), etc. [17]. Furthermore, there are various factors affecting the dispersibility such as the drying technique, the freezing procedure, drying and dispersion concentrations, and moisture content in the dried CNC [17,18].

Current processes to produce CNC powders involve the drying of CNC dispersions in water, using spray drying or freeze drying. These processes yield solid, hard, nonporous agglomerates where the nanoparticles adhere to each other due to strong, interparticle hydrogen bonds. Such agglomerates are difficult to disperse in polymer matrices by melt processing [19,20]. Recently, it was shown that highly porous CNC powders are obtained by using the spray freeze drying technique, which combines the advantages of atomization (spraying) and lyophilization (freeze drying) processes [21,22]. Spray freeze drying of CNC can provide larger surface area for polymer-CNC contact and interaction [23]. Overall, both dispersion quality and interfacial adhesion are enhanced with significant improvements in processing and mechanical characteristics of the nanocomposite [19,24]. 

The thermal stability of the PLA/CNC nanocomposites was found to be comparable to the PLA matrix and the presence of CNC did not compromise it [25]. Furthermore, the thermal stability of PLA nanocomposites incorporating various Montmorillonite (MMT) and organically modified MMT was studied using thermogravimetric analysis (TGA) and an increase in thermal stability was reported at a loading level of 3% (*w*/*w*) [26]. It was shown that initial degradation temperature of the PLA/PBSA nanocomposite containing 3 wt % of Cloisite 30B were superior as compared to that of PLA/PBSA blend. However, thermogravimetric analysis also revealed that thermal stability level of the PLA/PBSA nanocomposite containing 1 wt % of Cloisite 30B was only slightly better than that of PLA/PBSA blend due to the thermal instability of the surfactant present in the organoclay [27].

The crystallization of polylactic acid was reviewed extensively by Saeidlou and co-workers [28], while several references discussed chain structure [29], crystalline structure [30], glass transition temperature, melting temperature [31] and equilibrium melting point, as well as their crystallization kinetics. Heterogeneous nucleation using clay [32] and CNC [33] were also discussed. 

The present work reports on the preparation and characterization of melt processed PLA/PBSA blends and their nanocomposites incorporating spray freeze-dried MMT or CNC. The manuscript introduces results regarding the effects of using the nanoparticles on the morphology, dispersion, rheology, thermal and mechanical properties of the system. It also compares these characteristics to those of nanocomposites incorporating spray dried CNC and a commercially available organoclay. 

## 2. Experimental

### 2.1. Materials

Spray-dried cellulose nanocrystals (SD-CNC) were supplied by Forest Products Innovations (FPInnovations, Pointe-Claire, QC, Canada). They were used to prepare spray freeze-dried CNC (SFD-CNC). The average length and thickness of CNC, determined over 150 particles using atomic force microscopy (AFM) and transmission electron microscopy (TEM), were 171 ± 79.7 nm and 15.1 ± 5 nm, respectively [19]. Sodium montmorillonite (MMT) clay, with the commercial name Cloisite Na^+^ (cation exchange capacity (CEC) 92.6 meq/100g clay), was used to prepare spray freeze-dried MMT (SFD-MMT). Organoclay Cloisite 30B was also used in this study. It is an organically modified montmorillonite nanoclay with methyl, tallow, bis(2-hydroxyeethyl) quaternary ammonium (90 meq/100 g clay). Both MMT and C30B were purchased from Southern Clay Products, Gonzales, TX, USA, and they have basal spacings (d_001_), of 1.17 and 1.85 nm, respectively. The diols in Cloisite 30B can form strong bonds with the carbonyl (CO) group of PBSA [6]. All materials (PLA, PBSA, MMT and C30B) were dried under vacuum at 60 °C for 72 h prior to processing.

Poly(lactic acid) (PLA; 4032D), used in this study, was supplied by Natureworks, Minnetonka, MN, USA. It is a biodegradable polymer recommended for biaxially oriented films for packaging applications. Synthetic biodegradable poly[(butylene succinate)-*co*-adipate)] with mass density of 1.23 g/cm^3^ (PBSA; Bionolle 3001 M) was obtained from Showa Denko K.K., Tokyo, Japan. PBSA is a random copolyester which contains highly flexible macromolecules with excellent impact strength. It has relatively low production cost and good mechanical properties and processability compared to polyolefins [6]. An additive based on epoxy-functionalized PLA (CESA Extend OMAN698493, Clariant, Charlotte, NC, USA), hereafter called CESA, was used as chain extender to enhance both ductility and melt strength of the system. 

### 2.2. Preparation of FD-CNC, SFD-CNC, FD-MMT & SFD-MMT

Two grams of initially spray dried CNC or MMT were dispersed in 100 mL of reverse osmosis water using a shear mixer (digital ultra-turrax T25, IKA® Works, Inc., Staufen, Germany) and sonicated (model Q700, Qsonica, Newtown, CT, USA) for five minutes at room temperature. For producing freeze dried CNC (FD-CNC) and MMT (FD-MMT), the suspension was gently poured into liquid nitrogen and then freeze dried using a Labconco 2.5 L device (Labconco Corp, Kansas City, MO, USA), where the frozen droplets were lyophilized at −52 °C for two days to produce FD-CNC or FD-MMT. For SFD materials, the suspension was sprayed by a spray gun into liquid nitrogen, using a peristaltic pump. After the spray freezing step, the slurry containing the frozen droplets was transferred to the freeze dryer, where the frozen droplets were lyophilized at −52 °C for two days, yielding of spray freeze-dried CNC (SFD-CNC) or spray freeze-dried MMT (SFD-MMT).

### 2.3. Preparation of PLA Nanocomposites

PLA nanocomposites containing SFD-CNC, C30B or SFD-MMT were prepared using melt mixing via a Leistritz 18HP corotating twin-screw extruder (D 518 mm, L/D 540) equipped with eight controllable heating zones. The extruded strands were cooled using high-pressure air followed by water bath at room temperature and granulation in a pelletizer. A blend of PLA and PBSA was prepared, with composition (PLA/PBSA: 75/25) and 2 wt. % CESA based on PLA content (CPLA-PBSA). For the preparation of the nanocomposites, a masterbatch based on PLA was prepared by feeding PLA pellets through the main hopper and the powder (SFD-CNC, C30B or SFD-MMT) through the side feeder. The content of the filler (SFD-CNC, C30B or SFD-MMT) in the masterbatch was ~2.5 wt. %. The prepared pellets (masterbatch) were then dried in a vacuum oven at 60 °C for 12 h to remove any possible residual water. The prepared master batch was then used to prepare nanocomposites based on PLA/PBSA blends at a single filler concentration of 0.5 wt. %. All extrusion processes were performed at the screw speed of 100 rpm. The temperature distribution in the extruder was maintained at (from the feeder to the die): 170, 175, 175, 180, 180, 180, 180, 175 °C in the respective heating zones. Also, PLA, PLA with 2 wt. % chain extender (CPLA) and PBSA were extruded under the same processing conditions. All products were compression molded to obtain tensile and impact samples using a Carver hot press (Wabash, IN, USA). 

### 2.4. Characterization Methods

An FEI Inspect F-50 field emission scanning electron microscope (SEM) (FEI company, Hillsboro, OR, USA), operating at low accelerating voltages, was used to examine the morphology of MMT (10kV). The samples were coated with 4 nm thick platinum using an EM ACE600 Leica Microsystems high resolution sputter coater (Concord, ON, Canada) and placed directly without further surface treatment in the SEM chamber. SEM photomicrographs of MMT were obtained at 3.5 K magnifications. 

X-Ray diffraction (XRD) analyses were performed at room temperature using a Philips X’Pert PRO X-ray diffractometer (PAnalytical, Almelo, Netherlands) that generates a voltage of 50 kV and current 40 mA. The X-ray source was a tungsten filament tube with a Cu-target (K_α_=1.5418 Å). The diffraction angle 2θ was scanned from 1° to 40° for MMT clays at a step size of 0.05°. Bragg’s law was used to calculate the distance between the silicate layers. 

Specific surface areas of starting SD-MMT, FD-MMT, and SFD-MMT particles were determined using the multipoint N_2_ adsorption at −196 °C static volumetric technique, with a Micromeritic TriStar 3000 Surface Area and Porosity Analyzer (Micromeritics Instrument Corp., Norcross, GA, USA) in accordance with the BET (Brunauer–Emmett–Teller) method [34]. The samples (~150 mg) were outgassed under vacuum for 16 h at 80 °C for CNC. Adsorption/desorption of nitrogen was carried out in the relative pressure range of 0.01< P/P_o_ <1.00. Specific surface areas were determined from adsorption isotherms by applying the BET formalism in the relative pressure range of 0.05 < P/P_0_ < 0.25. Mesopore analysis was carried out using the Barrett, Joyner and Halenda (BJH) method based on application of the classical Kelvin equation for the estimation of the pore size [35].

For transmission electron microscopy (TEM), nanocomposites were trimmed to prepare a truncated pyramid section with a razor blade, to avoid the high pressure on the diamond knife. Then, they were ultra-microtomed at −70 °C (sample temperature) using a Reichert/Leica Ultra microtome to prepare samples with thickness of ~30 to ~50 nm. A cryo-wet 35° knife with DMSO/water bath was used to transfer the sections to 200-mesh copper grids. TEM micrographs were taken using a Jeol JEM-2100F Field Emission transmission electron microscope (Musashino, Tokyo, Japan) and recorded with a digital camera. 

All the samples were prepared for rheological measurements using a Carver press operated at 5 MPa and 180 °C for a period of 9 min heating (5 min preheating, 4 min heating under pressure) and 5 min cooling under pressure. A stress-controlled rheometer (Physica MCR 301, Anton Paar, Saint Laurent, QC, Canada) with parallel plate configuration was used to perform small amplitude oscillatory shear (SAOS) measurements over a frequency range from 0.1 to 100 rad/s at 180 °C. The gap was adjusted to 1.2 mm. The elongational viscosity was measured using a SER Universal Testing Platform in combination with ARES (Rheometric Scientific Inc., Piscataway, NJ, USA) rheometer at 180 °C. Dumbbell shape specimens for tensile tests (according to ASTM-D 638) were molded as described above. 

The specimens were microtomed from compression molded samples and placed in a differential scanning calorimeter (DSC-Pyris1, PerkinElmer, Waltham, MA, USA). Preliminary DSC experiments were conducted on C30B-CPLA and C30B-CPLA-PBSA using heat-cool-heat at 20 °C/min for each step. There were no crystallization peaks for both materials. Specimens were then heated from 20 to 200 °C at 20 °C/min and cooled to 20 °C at 10 °C/min and re-heated to 200 °C at 20 °C/min, followed by cooling back to 20 °C at 20 °C/min.

Polarized light microscopy (PLM) analysis was used to study the sizes of clay or CNC particles and of clusters in the polymer matrices. A BX50 Olympus polarized light microscope (Waltham, MA, USA) was used in conjunction with a hot-stage (model THMS600, Linkam Scientific Instruments, Surrey, UK) and equipped with CI93 temperature controller. Several 20-micron thick specimens were microtomed from tensile bars of all samples. The specimen was placed between two thin circular glass slices and placed on the hot stage, which was then heated to 200 °C to melt the specimen. Manual pressure was applied while allowing the specimen to cool from 200 °C to 50 °C, at ~50 °C/min (fast cool) to remove cutting stresses and previous thermo-mechanical history. Photographs were taken at room temperature and the specimen was then heated back to 200 °C to photograph the particles/clusters of the SFD-CNC, C30B clay and SFD-MMT. The specimen was allowed to cool back to room temperature and re-heated to 100 °C for cold crystallization or held at a temperature in the range of 106–120 °C to observe crystallization from the melt.

Mechanical testing of the nanocomposites was performed on dumbbell-shaped (gauge length: 25.4 mm, width: 1.9 mm, thickness: 3.2 mm) compression-molded samples. Tensile tests were conducted using an MTS Universal Tensile Testing machine (Eden Prairie, MN, USA) according to ASTM D 882-9. Specimens were properly aligned between the grips of the testing machine and the grips were tightened firmly to prevent any specimen slippage. Tensile strength (MPa), Young’s modulus (GPa) and percent elongation at break (%) were determined from the stress-strain diagrams. These properties were evaluated for a minimum of five specimens. The crosshead speed employed was 1 mm/min, based on a gauge length of 25.4 mm. The test temperature was 23 °C for all samples.

## 3. Results and Discussion

### 3.1. Morphology and Adsorption Analysis

The morphologies of spray dried, freeze dried and spray freeze-dried CNC were investigated in an earlier study [18] using SEM and XRD. Spray drying of CNC forms compact agglomerates of slightly deformed ellipsoidal shapes resulting from shrinkage due to capillary forces. Freeze dried CNC appears as slightly porous large irregular flakes resulting from the formation and growth of ice crystals, which causes the micro and nanoparticles to collide and aggregate. On the other hand, spray freeze drying of CNC from low concentration suspensions produces highly porous and large agglomerates. This is significantly influenced by the size distribution of suspension droplets as they freeze in liquid nitrogen after leaving the spray nozzle and attain a porous structure.

**Figure 1:**
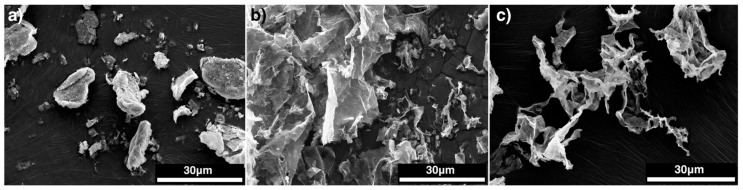
SEM micrographs of (**a**) SD-MMT (**b**) FD-MMT (**c**) SFD-MMT.

SEM photomicrographs of spray dried MMT, freeze-dried MMT and spray freeze-dried MMT taken at 3500 magnifications are given in Figure 1. SEM micrographs of MMT confirmed the developed platy surface texture of the clays after freeze drying and spray freeze-drying compared to the spray-dried original MMT, which showed dense agglomerates. Visually, FD-MMT were coarse agglomerates of irregular shapes ranging between 100 and 10,000 μm, which can cause issues during processing with polymers. SEM images showed clusters of small, individual, randomly oriented particles and some platy particles as well. SFD-MMT particles subjected to spray-freezing and then freeze-drying appeared as clusters of small, individual, randomly oriented flaky particles. As confirmed by BET surface area analyses and from the SEM micrographs, SFD-MMT has a porous structure compared to spray-dried and freeze-dried clay, likely due to the same reasons as for SFD-CNC. Spray-freeze-dried particles of both CNC [22] and MMT, with significantly enhanced porous structure, require less hydrodynamic forces, compared to spray-dried and freeze-dried particles, to obtain good dispersion in polymer nanocomposites.

**Figure 2:**
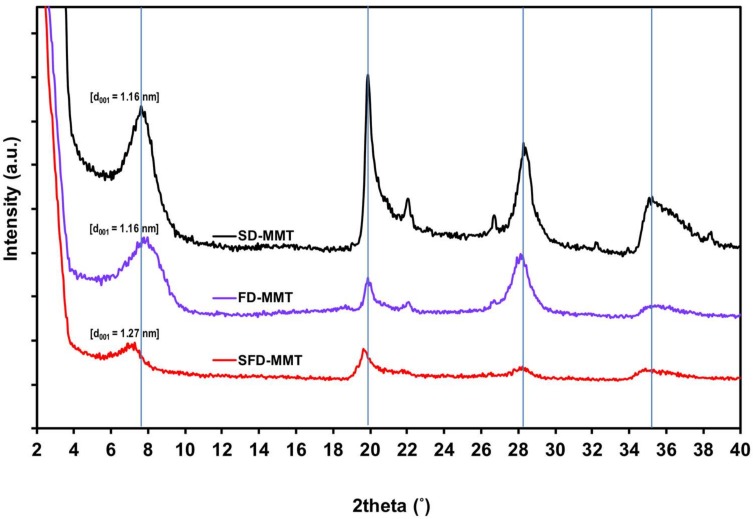
XRD diffractograms of MMT agglomerates: SD-MMT, FD-MMT and SFD-MMT.

The X-ray diffraction patterns for SD-MMT, FD-MMT and SFD-MMT clays and their corresponding basal spacing values are given in Figure 2. The analysis of XRD results confirmed the common characteristics of montmorillonite, with reflections relative to the planes (001) and (002) (M_001_ and M_002_). The interlayer distance of MMT 1.16 nm (2θ = 7.625°) did not change after freeze drying. On the other hand, SFD-MMT had a slightly larger interlayer spacing of 1.27 nm (2θ = 6.975°), an increase of 0.11 nm, and this can be attributed to the partial preservation of delaminated agglomerates in the 001 plane during the spraying/freezing step in liquid nitrogen.

**Figure 3:**
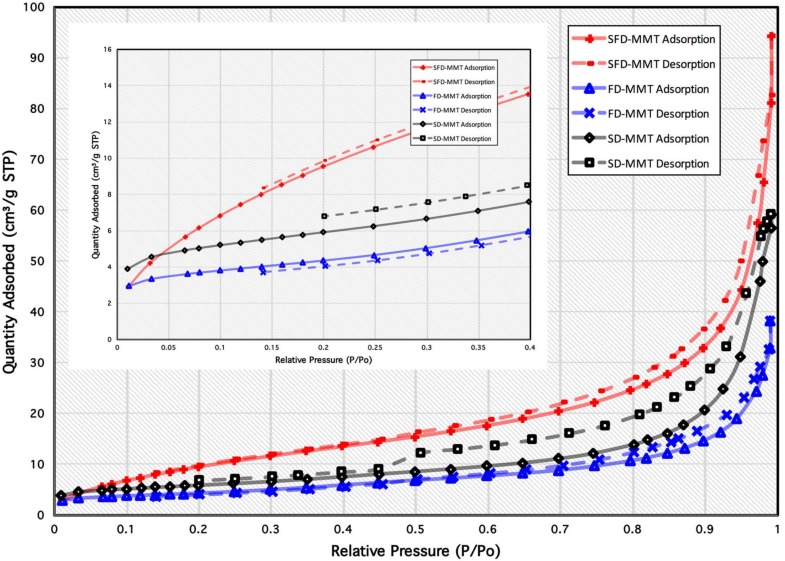
Nitrogen adsorption/desorption isotherms at 77 K of SD-MMT, FD-MMT and SFD-MMT particles.

The measurements of nitrogen adsorption/desorption isotherms were carried out in order to estimate the specific surface areas obtained as a result of the three drying methods in CNC (discussed elsewhere [18]) and MMT clay (Figure 3) particles. The data extracted from the isotherms of MMT samples are given in Table 1. The specific total surface area (S_BET_) calculated using the BET method in the range 0.05 < P/P_o_ < 0.2 for all MMT particles. All MMT clay isotherms can be classified as type IIB, with type H3 hysteresis loops indicating presence of mesopores, typical of nonrigid aggregates of plate-like particles possessing slit-shape pores [35]. Table 1 shows the specific total surface area (S_BET_) calculated using the BET method in the range 0.05 < P/P_o_ < 0.2 for all MMT clay particles. The BET surface area increases from 20.9 m^2^/g for SD-MMT to 40.4 m^2^/g for SFD-MMT prepared from spray freeze drying of low concentration MMT suspension which can be clearly seen in the house-of-cards like structures of SEM images. On the other hand, freeze drying of the same suspension resulted in the lowest value of S_BET_ (15.4 m^2^/g) in FD-MMT due to the absence of spraying, as in spray freeze drying process, which induces equal distribution of droplets when sprayed into liquid nitrogen.

### 3.2. Rheology Measurements

#### 3.2.1. Small Amplitude Oscillatory Shear (SAOS) Behavior and Morphological Characteristics

**Figure 4:**
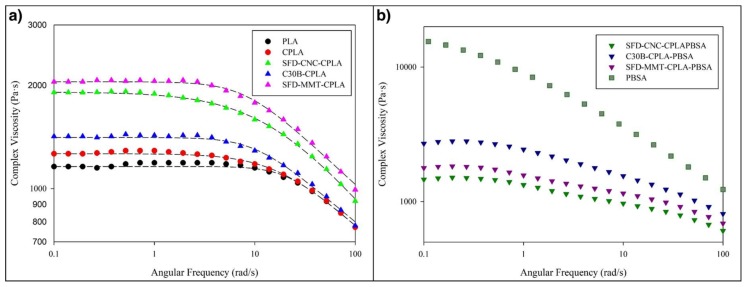
Complex viscosity of the (**a**) PLA, chain-extended PLA (CPLA), and CPLA nanocomposites with different nanoparticles and (**b**) corresponding CPLA-PBSA ternary nanocomposites.

The effect of spray freeze-dried cellulose nanocrystals (SFD-CNC) and montmorillonite (SFD-MMT) on the rheological properties of PLA nanocomposites and their corresponding blends with poly[(butylene succinate)-*co*-adipate] was investigated by performing small amplitude oscillatory shear measurements. Figure 4a shows the variation of complex viscosity as a function of frequency for PLA, chain-extended PLA (CPLA) and its corresponding nanocomposites with different nanoparticles. 

The Newtonian region at low shear rates and the pronounced shear thinning were observed for these samples; the second Newtonian region cannot be observed. The Carreau–Yasuda model (1) was fit quite well to the experimental data. The calculated zero-shear viscosity and pseudo-plasticity index values are presented in Table 2.

(1) $$\eta (\dot{\gamma })=\eta_{\infty }+\left(\eta_{o}-\eta_{\infty }\right){\left[{1+(\dot{\gamma }\lambda )}^{a}\right]}^{\frac{n-1}{a}},$$

All PLA nanocomposites had higher zero-shear viscosities and more pronounced shear thinning behavior when compared to the PLA and chain extended PLA (CPLA). Among the PLA nanocomposites, pseudo-plasticity was more pronounced for the samples incorporating SFD-CNC (pseudo-plasticity index of 0.65). 

**Figure 5:**
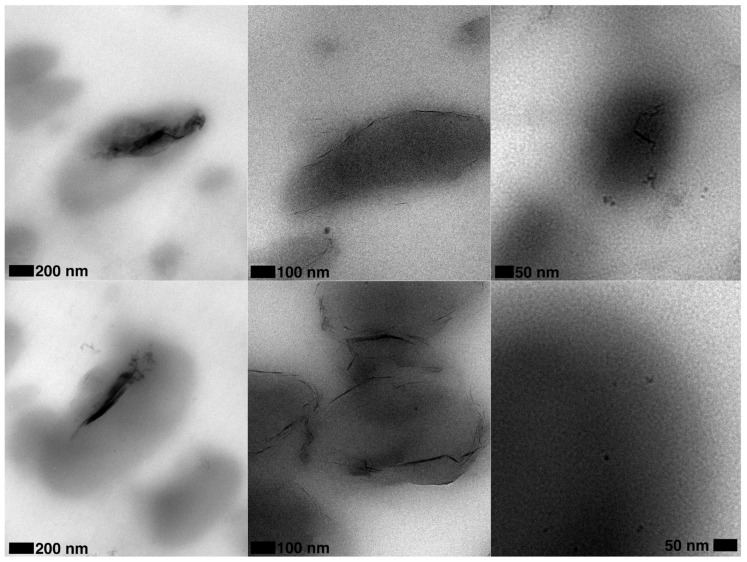
TEM micrographs of (**left**) SFD-MMT-CPLA-PBSA; (**middle**) C30B-CPLA-PBSA and (**right**) SFD-CNC-CPLA-PBSA.

The use of Cloisite 30B (C30B) is more effective than chemically unmodified spray freeze-dried nanoparticles (SFD-CNC and SFD-MMT) in changing the rheological properties of CPLA-PBSA ternary nanocomposites (Figure 4b) which is opposite to the trend observed for the PLA nanocomposites. This may be explained by the localization of the nanoparticles in CPLA-PBSA ternary nanocomposites caused by surface energy factors. These factors cause the modified montmorillonite platelets of C30B to be located at the phase interface in C30B-CPLA-PBSA ternary nanocomposite. TEM micrographs support this prediction and show that the PBSA droplets are surrounded by C30B layers while the SFD-MMT layers are located inside the PBSA droplets (Figure 5). Consequently, the local concentration of the chain extender in the PLA phase of C30B-CPLA-PBSA would be more than that of SFD-CNC-CPLA-PBSA and SFD-MMT-CPLA-PBSA due to the barrier effect of clay layers [36]. More details regarding the selective localization of the nanoparticles, their surface energies and wetting coefficients in the ternary systems could be found in previous publications [6,24,36,37,38]. 

**Figure 6:**
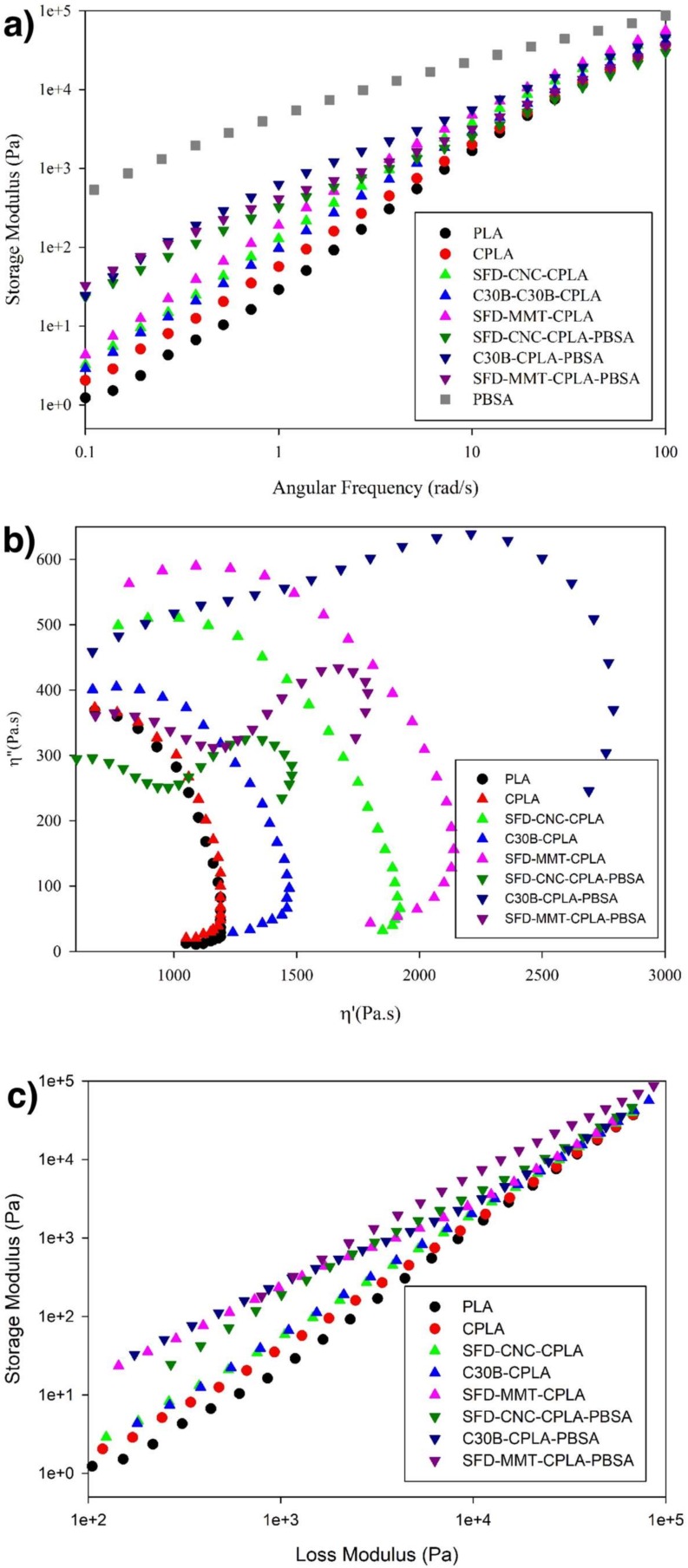
(**a**) Storage modulus, (**b**) Cole–Cole plot and (**c**) modified Cole–Cole plot of the pure components, PLA nanocomposites and their corresponding PLA-PBSA blends and nanocomposites.

Storage modulus as a function of angular frequency, *ω*, also provides insight about the morphological features of the materials and their rheological response. As seen in Figure 6a, Storage modulus was proportional to *ω*^2^ at low frequencies for PLA, which is the typical behavior for linear polymers. Chain extension reaction caused a slight deviation from terminal behavior for CPLA. Higher storage modulus and smaller slope were observed in the low frequency region by incorporation of nanoparticles, as compared to PLA and CPLA. Higher elasticity in the low frequency region and lower slope in the terminal zone were found for the PLA nanocomposites prepared based on spray freeze-dried nanoparticles as compared to the chemically modified MMT (C30B). The deviation from the terminal zone behavior was more pronounced for SFD-MMT-CPLA due to the improved dispersion of SFD-MMT nanoparticles which contributes to higher reinforcement of the PLA matrix.

The stiffness of C30B is about 3 times that of CNC [39]. However, elastic properties of SFD-CNC-CPLA were slightly higher than those of C30B-CPLA, as a result of the enhanced dispersion of the highly porous CNC particles and their interactions with the polymer.

The storage modulus of C30B-CPLA-PBSA ternary nanocomposite was higher than those of CPLA-PBSA ternary nanocomposites prepared based on spray freeze-dried nanoparticles because of the higher extent of chain extension reaction. The barrier role of C30B layers that controls the local concentration of chain extender in the PLA phase and its effect on the extent of chain extension reaction was discussed in detail in a previous study [36]. 

Furthermore, The Cole–Cole plot (Figure 6b) and modified Cole–Cole plot (Figure 6c) were used to indicate the differences in the dispersion levels of nanoparticles in PLA nanocomposites and microstructure of the PLA-PBSA ternary nanocomposites. A concave Cole–Cole curve close to a semicircle, which is a typical curve for linear polymers, was observed for PLA and CPLA. The extent of the deviation from the semicircular shape was more obvious for nanocomposites prepared based on sonicated spray freeze-dried nanoparticles than for PLA and CPLA and C30B-CPLA due to the better dispersion of the SFD particles as indicated above. 

**Figure 7:**
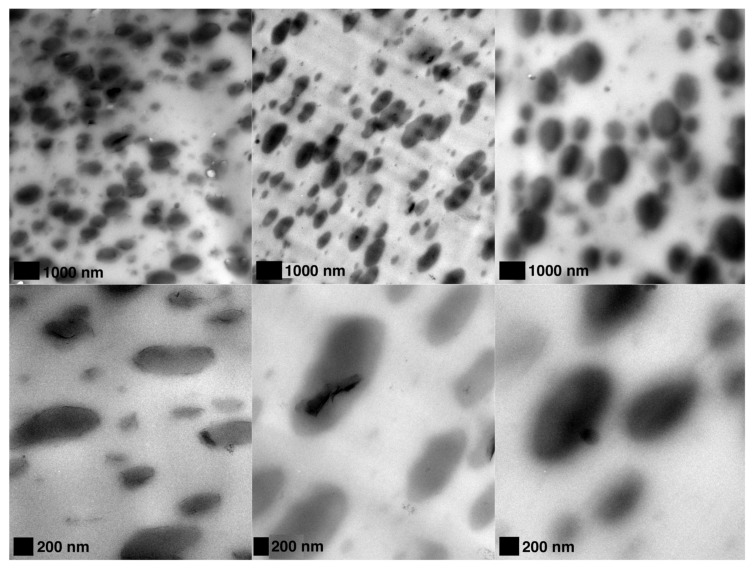
TEM micrographs of (**left**) C30B-CPLA-PBSA (**middle**) SFD-MMT-CPLA-PBSA and (**right**) SFD-CNC-CPLA-PBSA.

The rheological responses of the CPLA-PBSA ternary nanocomposites were quite different. The characteristic of the Cole–Cole plot for homogeneous polymeric blends is the existence of only one circular arc. After the phase temperature transition of a system, a tail develops and forms a second arc on the right-hand side of the first arc [40]. The morphological studies agree with these results and they suggest that the droplet-matrix morphology is applicable to all the prepared CPLA-PBSA ternary nanocomposites as seen in Figure 7. The ternary blend based on C30B showed significant elasticity as compared to the ternary nanocomposites containing spray freeze-dried nanoparticles. However, the effectiveness of the highly porous nanoparticles in raising the elasticity of PLA nanocomposites is higher than that of C30B (Figure 6).

#### 3.2.2. Elongational Viscosity Behavior

**Figure 8:**
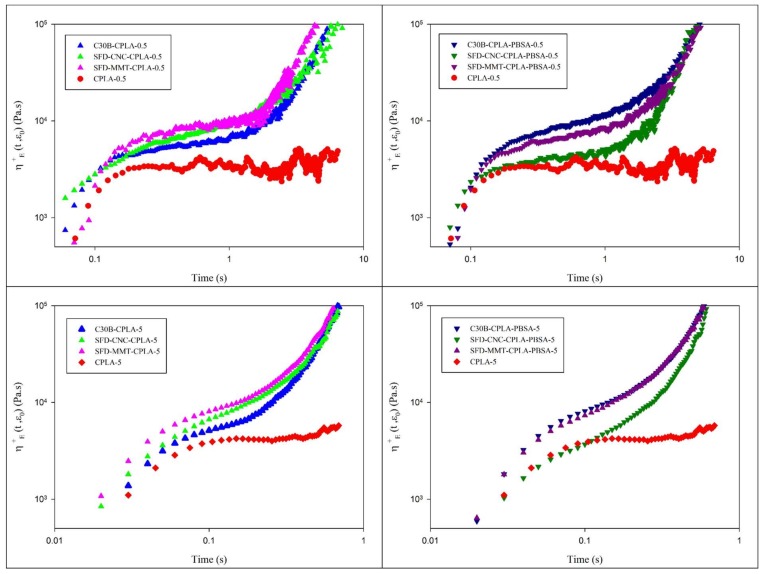
Elongational viscosity of the CPLA nanocomposites (**left**) and CPLA-PBSA ternary nanocomposites (**right**) at different Hencky strain rate of 0.5 s^−1^ and 5 s^−1^.

Unmodified PLA exhibits low elongational viscosity and melt strength which has limited its use in many important applications such as film blowing, blow molding, thermoforming, and foaming. Different approaches have been reported to enhance the elongational viscosity and melt strength of PLA systems such as the use of chain extender in reactive extrusion process [36], the use of chemically modified nanoclay in combination with chain extender [41] and by addition of microcrystalline cellulose-graft-polylactic acid copolymer [42]. The effect of spray freeze-dried nanoparticles on extensional rheology of PLA nanocomposites and PLA-PBSA ternary nanocomposites were investigated and compared to that of PLA at different Hencky strain rates. Elongational viscosity can be improved by blending with PBSA and increasing the extent of chain extension reaction. Moreover, this can be achieved or enhanced by the effective dispersion of appropriate nanoparticles, which may reduce the rate of chain disentanglement. On the other hand, thermal degradation could have a negative influence on melt strength. As seen in Figure 8, CPLA does not exhibit strain hardening behavior. It shows the lowest elongational viscosity and a noisy curve, particularly at low Hencky rate, due to the thermal degradation of PLA during extrusion. On the other hand, chain extended PLA nanocomposites displayed the interesting well-defined strain-hardening behavior. Remarkable improvement in elongational viscosity was observed for the samples prepared based on SFD-MMT especially at higher Hencky strain rate. Depending on the nanoparticle used, the magnitude of elongational viscosity and the intensity of the strain hardening behavior were altered. Based on previous studies [36,41], the same level of chain extension reaction is expected for C30B-CPLA, SFD-MMT-CPLA and SFD-CNC-CPLA. Therefore, the observed differences were attributed to differences among the dispersion levels and interfacial characteristics of the nanoparticles used. Spray freeze-dried nanoparticles showed promising contribution to increasing the melt strength of the resulting nanocomposites as compared to C30B.

As mentioned before, the localization of the nanoparticles due to the surface energy considerations and their shapes affect the efficiency of the chain extender. Therefore, the higher melt strength of the C30B-CPLA-PBSA and SFD-CNC-CPLA-PBSA, as compared to that of SFD-MMT-CPLA-PBSA, is attributed to the barrier effect of montmorillonite layers at the interface between PLA and PBSA. It increases the local concentration of the chain extender within the PLA phase and leads to longer chains/branches.

#### 3.2.3. Calorimetric Crystallization Properties

**Figure 9:**
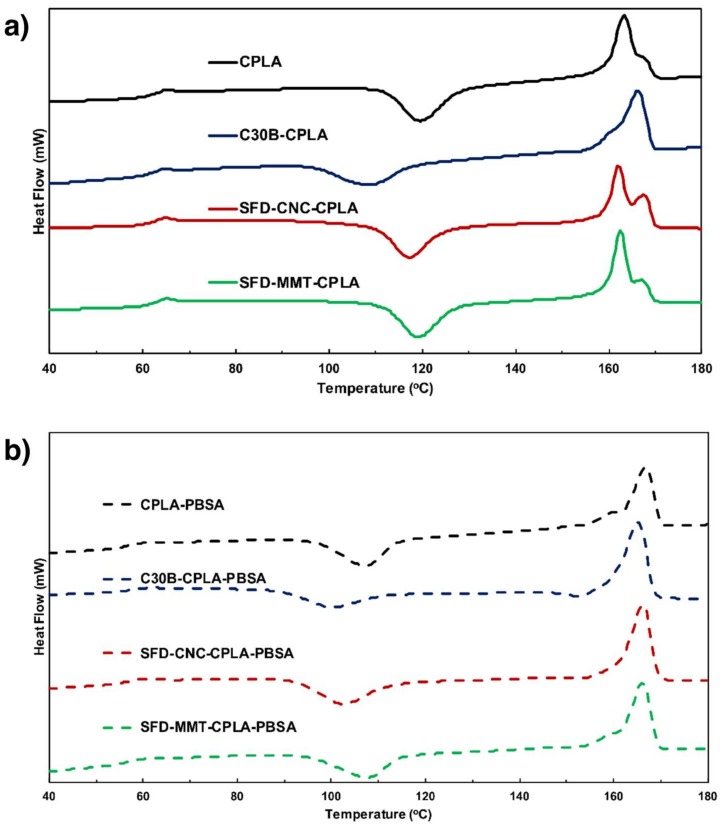
DSC endotherms from second heating experiments of (**a**) CPLA and (**b**) CPLA-PBSA compositions.

The values of onset of crystallization temperature T_c-onset_ and the heat of crystallization ΔH_C_ during cooling at 10 °C/min, the onset of cold-crystallization temperature T_cc-onset_, the heat of fusion ΔH_m_ and the heat of cold-crystallization resulting from the re-arrangement of macromolecules during the second heating ΔH_cc_ in J/(g of PLA) are shown in Table 3. The endotherm for CPLA and CPLA-PBSA compositions are shown in Figure 9. The degree of crystallinity X_C_ is obtained by dividing the value of ΔHm of each sample by the value of 106 J/g for ΔHm* of 100% PLA as reported by Sarasua and co-workers [43]. All samples showed partial crystallization during cooling at 10 °C/min in the temperature range of 110 to 70 °C and resulted in small cold crystallization upon second heating. 

C30B seems to provide more effective nucleation sites (earlier T_cc-onset_) in both CPLA and CPLA-PBSA, followed by SFD-CNC and SFD-MMT. C30B present in C30B-CPLA also crystallized to form lamellae that exhibited only a predominant melting peak at 166.25 °C (α crystal form) in CPLA, while the CPLA showed a predominant melting peak at 163.25 °C (imperfect or smaller lamellae α crystal form). Only a single melting peak around 166 °C was observed for the CPLA-PBSA series.

CPLA exhibited a T_g_ of 61.2 °C and the melting peak at 163.3 °C and heat of fusion of 36.7 J/g. The CPLA-PBSA showed a T_g_ of 55.5 °C due to the presence of PBSA with T_g_ at −44 °C [44] indicating that there were regions of CPLA that blended with PBSA. PBSA also affected the heat of fusion of CPLA from 36.7 J/g to 35.1 J/g. CPLA-PBSA with 0.5% SFD-MMT and 0.5% SFD-CNC were quite similar with lowering the T_g_ of CPLA from 59 °C to 54 °C due to the presence of PBSA.

CPLA with 0.5% SFD-MMT and 0.5% SFD-CNC showed heat of fusion of 37.7 J/g with double T_m_ peaks at 162.5 and 167.7 °C, while those of CPLA-PBSA with 0.5% SFD-MMT and 0.5% SFD-CNC showed T_m_ 166.2 °C and heat of fusion of 35 to 37 J/(g of PLA).

The nanoparticles of MMT and CNC are well known to provide heterogeneous sites for the nucleation of polymers [24]. SFD-CNC was more effective in providing heterogeneous sites for the cold crystallization of CPLA (95% complete). SFD-MMT in both CPLA and CPLA-PBSA series showed the highest T_cc_-onset, while that of C30B in CPLA and CPLA-PBSA were the lowest. Thus, the CPLA-C30B was expected to show the smallest PLA spherulites during the cold crystallization. SFD-MMT and SFD-CNC composites with PBSA showed some hindrance effects on the cold crystallization of the PLA resulting in a much lower value of ΔH_cc_ (66.3–83% of ΔH_m_), compared to the PLA without PBSA (86.7–95%).

### 3.3. Particle and Cluster Size Distribution Study

**Figure 10:**
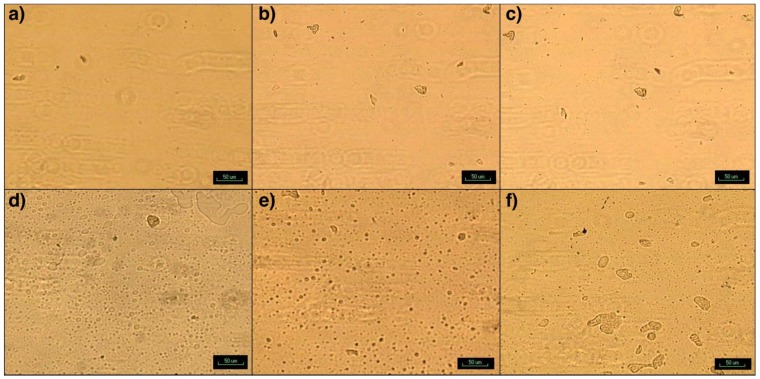
Photomicrographs of spherulitic structure of (**a**) C30B-CPLA, (**b**) SFD-CNC-CPLA, (**c**) SFD-MMT-CPLA, (**d**) C30B-CPLA-PBSA, (**e**) SFD-CNC-CPLA-PBSA and (**f**) SFD-MMT-CPLA-PBSA.

While the particles and clusters can be observed under parallel polarizers at room temperature, they could be best seen at 200 °C under crossed polarized light, when the matrix is completely melted (Figure 10). This is due to the birefringence of SFD-MMT and SFD-CNC. PBSA appears as a second phase with a semi-round border at the interphase, while the clusters of Clay, SFDC and SFDNCC appear as irregular shaped objects and they exhibit the birefringence characteristics at 200 °C as bright objects or the Maltese cross pattern, depending on the complexity of the particle arrangement inside the cluster. Furthermore, the nanoparticles act as heterogeneous sites for crystallization of the PLA, when held at a constant temperature in the range of 100–130 °C [28], which is outside the scope of this study.

**Figure 11:**
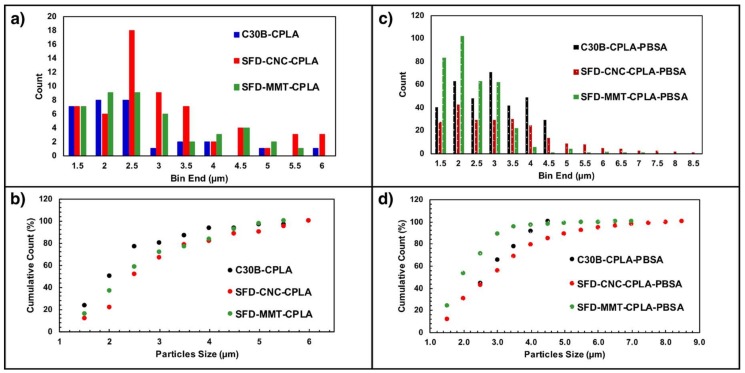
Particle size distribution of C30B, SFD-CNC and SFD-MMT in CPLA (**a**,**b**) and CPLA-PBSA (**c**,**d**) samples.

The particle size distribution for each specimen was obtained using ImageJ (Figure 11). The presence of PBSA improved the dispersion quality, resulting in ~5 times the number of particles for the case of CPLA without the PBSA. PBSA also trends to produce more of the finer particles with “equivalent” diameter of less than 2.0 microns. The blending of PBSA with PLA improved the dispersion and distribution of the nanoparticles of C30B, SFD-MMT and SFD-CNC. The dispersion of these nanoparticles may affect the crystallization of PLA, depending on the interfacial tension (interaction) of the nanoparticles which provide heterogenous nucleation sites for crystallization and eventually affecting the mechanical properties.

### 3.4. Mechanical Properties

**Figure 12:**
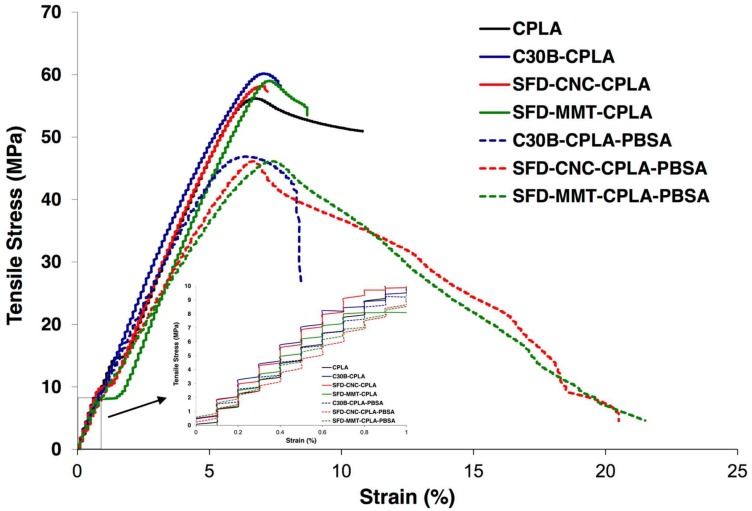
Stress–strain curve of the PLA nanocomposites and PLA-PBSA ternary nanocomposites.

It is well known that a good quality of dispersion and/or exfoliation of nanofillers in the polymer matrix is required to enhance the mechanical properties. Different approaches have been taken to enhance the dispersion quality of nanoparticles, and consequently the mechanical properties of the nanocomposites, such as the use of chemical modifiers for montmorillonite layers [45] and grafting by ring opening polymerization of l-Lactide initiated from hydroxyl group for CNC [46]. In this work, the authors investigated the potential of the spray freeze drying technique to enhance the dispersion level without using chemical modifiers or other methods. The tensile stress-strain curves and the corresponding mechanical results are presented in Figure 12 and Table 4. The brittle behavior and rupture at low strain of CPLA as a result of the crosslinking reaction and its corresponding nanocomposites are evident. The modulus of the SFD-MMT-CPLA was slightly higher than that of other samples. The modulus of the nanocomposites prepared by SFD-CNC was at the same level as for C30B, reflecting the influence of the presence of a larger number of nanoparticles in the spray freeze-dried systems. While stable necking was not observed for the PLA/PBSA nanocomposites, a noteworthy increase in the strain at break was observed as compared to PLA and PLA nanocomposites.

## 4. Conclusions

PLA as a biosource, biocompatible and biodegradable polymer is a likely candidate for use in many applications. However, significant shortcomings in its processing behavior and mechanical properties limit its suitability in a variety of applications. Thus, a variety of approaches have been used to overcome some of these limitations. Typically, blending with other biopolymers, such as PBSA, the use of chain extenders, and the incorporation of nanoparticles, have been used individually or in combination for this purpose. The present study evaluates the effects of some of these modifications. Emphasis is placed on the following factors, both individually and in combination: the use of chain extender, the blending with PBSA, the use of montmorillonite clay (modified or unmodified) and cellulose nanocrystals (CNC) as fillers to form nanocomposites.

The results indicate that the comprehensive approach utilizing the nanocomposites of the chain extended PLA-PBSA blend yields significant improvement. Obviously, it would be desirable for many applications to employ CNC, in order to maintain the biofriendly character of the nanocomposites. In this case, it is shown that permeable spray freeze-dried cellulose nanocrystal (SFD-CNC) particles provide satisfactory enhancements in rheological and mechanical properties. Similarly, it is shown that spray freeze-dried unmodified clay yields results comparable to those of chemically modified clay. Thus, spray freeze drying of clay is a viable approach to producing nanoparticles suitable for the production of nanocomposites without chemical modifications.

## Figures and Tables

**Table 1:** Numerical results derived from the BET and BJH methods of the N_2_ adsorption–desorption isotherms for MMT samples.

Material	BET	BJH Adsorption			BJH Desorption		
-	S_BET_ _(m_^2^_/g)_	S_BJH-Ads. (m_^2^_/g)_	V_P-Ads._ _(c.c./g)_	r_P-Ads._ _(nm)_	S_BJH-Des. (m_^2^_/g)_	V_P-Des._ _(c.c./g)_	r_P-Des._ _(nm)_
**SD-MMT**	20.9	26.22	0.0898	20.2	17.78	0.0916	13.9
**FD-MMT**	15.4	15.14	0.0585	15.5	17.14	0.0598	13.9
**SFD-MMT**	40.4	43.62	0.1473	13.5	45.34	0.1472	13.0

S_BET_: BET surface area; S_BJH-Ads._: BJH adsorption cumulative surface area of pores between 1.7 nm and 300 nm width; V_P-Ads._: BJH adsorption cumulative volume of pores between 1.7 nm and 300 nm width, r_P-Ads._: BJH adsorption average pore width (4V/A), S_BJH-Des._: BJH desorption cumulative surface area of pores between 1.7 nm and 300 nm width, V_P-Des._: BJH desorption cumulative volume of pores between 1.7 nm and 300 nm width, r_P-Des._: BJH desorption average pore width (4V/A).

**Table 2:** Carreau–Yasuda model’s parameters for PLA, CPLA and corresponding nanocomposites.

Sample	(n) Pseudo-plasticity Index	(a) Carreau Constant	(*η*_0_) Zero-shear Viscosity (Pa·s)	(λ) RelaxationTime (s)
**PLA**	0.76	2.6	1160	0.048
**CPLA**	0.74	1.5	1264	0.057
**SFD-CNC-CPLA**	0.65	1.0	1912	0.070
**C30B-CPLA**	0.73	2.1	1409	0.099
**SFD-MMT-CPLA**	0.72	2.0	2048	0.126

**Table 3:** DSC data for all PLA compositions.

Material	T_c-onset_	ΔH_c_	T_g_	T_cc-onset_	ΔH_cc_	T_m_ Peak	ΔH_m_	X_c_
**-**	[°C]	[J/g]	[°C]	[°C]	[J/g]	[°C]	[J/g]	[%]
**CPLA**	112.62	-	61.15	112.02	−35.55	163.25	36.71	34.6
**C30B-CPLA**	108.2	−2.20	60.63	97.87	−31.04	166.25	35.31	33.3
**SFD-CNC-CPLA**	107.6	−0.78	60.45	109.97	−35.93	162.24/167.91	37.69	35.6
**SFD-MMT-CPLA**	110.4	−0.72	58.88	111.66	−32.79	162.55/167.56	37.80	35.7
**CPLA-PBSA**	106.7	−1.33	55.47	96.75	−31.15	166.87	35.13	33.1
**C30B-CPLA-PBSA**	107.6	−7.00	55.95	89.89	−26.10	165.21	39.36	37.1
**SFD-CNC-CPLA-PBSA**	109.3	−2.32	53.63	92.68	−30.92	166.22	37.18	35.1
**SFD-MMT-CPLA-PBSA**	112.0	−2.28	55.10	97.15	−26.94	166.24	35.28	33.3

**Table 4:** Mechanical results for PLA compositions.

Material	Tensile Strength (MPa)	Young’s Modulus (MPa)	% Elongation at Break
**CPLA**	51.5 ± 1.3	1029.2 ± 0.01	11.5 ± 0.2
**C30B-CPLA**	56.7 ± 1.5	1099.4 ± 0.03	6.8± 0.8
**SFD-CNC-CPLA**	56.8 ± 1.4	1057.2 ± 0.02	6.9 ± 0.2
**SFD-MMT-CPLA**	59.5 ± 0.6	1156.6 ± 0.03	8.4 ± 0.4
**C30B-CPLA-PBSA**	46.4 ± 0.8	966.1 ± 0.00	8.5 ± 0.2
**SFD-CNC-CPLA-PBSA**	45.4 ± 0.9	930.7 ± 0.03	21.1 ± 0.3
**SFD-MMT-CPLA-PBSA**	46.0 ± 0.1	894.9 ± 0.06	22.2 ± 0.7

## References

[1] Iwatake A., Nogi M., Yano H. (2008). Cellulose nanofiber-reinforced polylactic acid. Compos. Sci. Technol..

[2] Sawyer D.J. (2003). Bioprocessing—No Longer a Field of Dreams. Macromol. Symp..

[3] Rasal R.M., Janorkar A.V., Hirt D.E. (2010). Poly(lactic acid) modifications. Prog. Polym. Sci..

[4] Lin S., Guo W.N., Chen C.Y., Ma J.L., Wang B.B. (2012). Mechanical properties and morphology of biodegradable poly(lactic acid)/poly(butylene adipate-co-terephthalate) blends compatibilized by transesterification. Mater. Des..

[5] Vroman I., Tighzert L. (2009). Biodegradable polymers. Materials.

[6] Eslami H., Kamal M.R. (2012). Elongational rheology of biodegradable poly(lactic acid)/poly[(butylene succinate)-co-adipate] binary blends and poly(lactic acid)/poly[(butylene succinate)-co-adipate]/clay ternary nanocomposites. J. Appl. Polym. Sci..

[7] Ojijo V., Ray S.S., Sadiku R. (2012). Effect of Nanoclay Loading on the Thermal and Mechanical Properties of Biodegradable Polylactide/Poly[(butylene succinate)-co-adipate] Blend Composites. ACS Appl. Mater. Interfaces.

[8] Gui Z.Y., Wang H.R., Gao Y., Lu C., Cheng S.J. (2012). Morphology and melt rheology of biodegradable poly(lactic acid)/poly(butylene succinate adipate) blends: Effect of blend compositions. Iran Polym. J..

[9] Boujemaoui A., Mongkhontreerat S., Malmstrom E., Carlmark A. (2015). Preparation and characterization of functionalized cellulose nanocrystals. Carbohydr. Polym..

[10] Iyer K.A., Schueneman G.T., Torkelson J.M. (2015). Cellulose nanocrystal/polyolefin biocomposites prepared by solid-state shear pulverization: Superior dispersion leading to synergistic property enhancements. Polymer.

[11] Favre H., Lagaly G. (1991). Organo-bentonites with quaternary alkylammonium ions. Clay Miner..

[12] Abdallah W., Yilmazer U. (2013). Preparation and characterization of thermally stable phosphonium organoclays and their use in poly(ethylene terephthalate) nanocomposites. J. Appl. Polym. Sci..

[13] Sapkota J., Jorfi M., Weder C., Foster E.J. (2014). Reinforcing Poly(ethylene) with Cellulose Nanocrystals. Macromol. Rapid Commun..

[14] Ma L., Zhang Y., Wang S. (2015). Preparation and characterization of acrylonitrile-butadiene-styrene nanocomposites reinforced with cellulose nanocrystal via solution casting method. Poly. Compos..

[15] Voronova M.I., Surov O.V., Guseinov S.S., Barannikov V.P., Zakharov A.G. (2015). Thermal stability of polyvinyl alcohol/nanocrystalline cellulose composites. Carbohydr. Polym..

[16] Beuguel Q., Tavares J.R., Carreau P.J., Heuzey M.-C. (2018). Ultrasonication of spray- and freeze-dried cellulose nanocrystals in water. J. Colloid Interface Sci..

[17] Beck S., Bouchard J., Berry R. (2012). Dispersibility in Water of Dried Nanocrystalline Cellulose. Biomacromolecules.

[18] Abdallah W., Kamal M.R. (2018). Influence of process variables on physical characteristics of spray freeze dried cellulose nanocrystals. Cellulose.

[19] Khoshkava V., Kamal M.R. (2013). Effect of surface energy on dispersion and mechanical properties of polymer/nanocrystalline cellulose nanocomposites. Biomacromolecules.

[20] Zhang W., He X., Li C., Zhang X., Lu C., Zhang X., Deng Y. (2014). High performance poly (vinyl alcohol)/cellulose nanocrystals nanocomposites manufactured by injection molding. Cellulose.

[21] Kamal M., Khoshkava V. (2017). Spray Freeze-Dried Nanoparticles and Method of Use Thereof. Google Patents.

[22] Khoshkava V., Kamal M.R. (2014). Effect of drying conditions on cellulose nanocrystal (CNC) agglomerate porosity and dispersibility in polymer nanocomposites. Powder Technol..

[23] Khoshkava V., Kamal M.R. (2014). Effect of Cellulose Nanocrystals (CNC) Particle Morphology on Dispersion and Rheological and Mechanical Properties of Polypropylene/CNC Nanocomposites. ACS Appl. Mater. Interfaces.

[24] Kamal M.R., Khoshkava V. (2015). Effect of cellulose nanocrystals (CNC) on rheological and mechanical properties and crystallization behavior of PLA/CNC nanocomposites. Carbohydr. Polym..

[25] Sullivan E.M., Moon R.J., Kalaitzidou K.J.M. (2015). Processing and characterization of cellulose nanocrystals/polylactic acid nanocomposite films. Sci. J..

[26] Krishnamachari P., Zhang J., Lou J., Yan J., Uitenham L. (2009). Biodegradable Poly(Lactic Acid)/Clay Nanocomposites by Melt Intercalation: A Study of Morphological, Thermal, and Mechanical Properties. Int. J. Polym. Anal. Charact..

[27] Bhatia A., Gupta R.K., Bhattacharya S.N., Choi H.J.J. (2009). An investigation of melt rheology and thermal stability of poly (lactic acid)/poly (butylene succinate) nanocomposites. J. Appl. Polym. Sci..

[28] Saeidlou S., Huneault M.A., Li H., Park C.B. (2012). Poly(lactic acid) crystallization. Prog. Polym. Sci..

[29] Auras R.A., Lim L.-T., Selke S.E., Tsuji H. (2011). Poly (Lactic Acid): Synthesis, Structures, Properties, Processing, and Applications.

[30] Carrasco F., Pagès P., Gámez-Pérez J., Santana O., Maspoch M.L. (2010). Processing of poly (lactic acid): Characterization of chemical structure, thermal stability and mechanical properties. Polym. Degrad. Stab..

[31] Petersson L., Kvien I., Oksman K. (2007). Structure and thermal properties of poly (lactic acid)/cellulose whiskers nanocomposite materials. Compos. Sci. Technol..

[32] Ogata N., Jimenez G., Kawai H., Ogihara T. (1997). Structure and thermal/mechanical properties of poly(l-lactide)-clay blend. J. Polym. Sci. Pol. Phys..

[33] Pei A., Zhou Q., Berglund L.A. (2010). Functionalized cellulose nanocrystals as biobased nucleation agents in poly (l-lactide)(PLLA)–Crystallization and mechanical property effects. Compos. Sci. Technol..

[34] Brunauer S., Emmett P.H., Teller E. (1938). Adsorption of gases in multimolecular layers. J. Am. Chem. Soc..

[35] Rouquerol F.R.J.S.K.S.W. Adsorption by Powders and Porous Solids Principles, Methodology, and Applications. http://site.ebrary.com/id/10190030.

[36] Mirzadeh A., Ghasemi H., Mahrous F., Kamal M.R. (2015). Reactive extrusion effects on rheological and mechanical properties of poly(lactic acid)/poly[(butylene succinate)-co-adipate]/epoxy chain extender blends and clay nanocomposites. J. Appl. Polym. Sci..

[37] Calderon J.U., Kamal M.R. (2007). Influence of surface energy on dispersion and flexural properties of PS/phosphonium organoclay nanocomposites. Annu. Technol. Conf. Soc. Plast. Eng..

[38] Kamal M.R., Calderon J.U., Lennox R.B. (2009). Surface energy of modified nanoclays and its effect on polymer/clay nanocomposites. J. Adhes. Sci. Technol..

[39] Manevitch O.L., Rutledge G.C. (2004). Elastic Properties of a Single Lamella of Montmorillonite by Molecular Dynamics Simulation. J. Phys. Chem. B.

[40] Ajji A., Choplin L., Prud’homme R.E. (1988). Rheology and phase separation in polystyrene/poly(vinyl methyl ether) blends. J. Polym. Sci. Pol. Phys..

[41] Eslami H., Kamal M.R. (2013). Effect of a chain extender on the rheological and mechanical properties of biodegradable poly(lactic acid)/poly[(butylene succinate)-co-adipate] blends. J. Appl. Polym. Sci..

[42] Hua S., Chen F., Liu Z.-Y., Yang W., Yang M.-B. (2016). Preparation of cellulose-graft-polylactic acid via melt copolycondensation for use in polylactic acid based composites: Synthesis, characterization and properties. Rsc. Adv..

[43] Sarasua J.-R., Prud’homme R.E., Wisniewski M., Le Borgne A., Spassky N. (1998). Crystallization and Melting Behavior of Polylactides. Macromolecules.

[44] Lee S., Lee J.W. (2005). Characterization and processing of biodegradable polymer blends of poly (lactic acid) with poly (butylene succinate adipate). Korea-Aust. Rheol. J..

[45] Charlon S., Follain N., Dargent E., Soulestin J., Sclavons M., Marais S. (2016). Poly[(butylene succinate)-co-(butylene adipate)]-Montmorillonite Nanocomposites Prepared by Water-Assisted Extrusion: Role of the Dispersion Level and of the Structure-Microstructure on the Enhanced Barrier Properties. J. Phys. Chem. C.

[46] Lizundia E., Fortunati E., Dominici F., Vilas J.L., León L.M., Armentano I., Torre L., Kenny J.M. (2016). PLLA-grafted cellulose nanocrystals: Role of the CNC content and grafting on the PLA bionanocomposite film properties. Carbohydr. Polym..

